# Immunosuppression mediated by myeloid-derived suppressor cells (MDSCs) during tumour progression

**DOI:** 10.1038/s41416-018-0333-1

**Published:** 2018-11-09

**Authors:** Christopher Groth, Xiaoying Hu, Rebekka Weber, Viktor Fleming, Peter Altevogt, Jochen Utikal, Viktor Umansky

**Affiliations:** 1 Skin Cancer Unit, German Cancer Research Center (DKFZ), Heidelberg, Germany; 2 Department of Dermatology, Venereology and Allergology, University Medical Center Mannheim, Ruprecht-Karl University of Heidelberg, Mannheim, Germany

**Keywords:** Immunosurveillance, Immunosuppression

## Abstract

Under steady-state conditions, bone marrow-derived immature myeloid cells (IMC) differentiate into granulocytes, macrophages and dendritic cells (DCs). This differentiation is impaired under chronic inflammatory conditions, which are typical for tumour progression, leading to the accumulation of IMCs. These cells are capable of inducing strong immunosuppressive effects through the expression of various cytokines and immune regulatory molecules, inhibition of lymphocyte homing, stimulation of other immunosuppressive cells, depletion of metabolites critical for T cell functions, expression of ectoenzymes regulating adenosine metabolism, and the production of reactive species. IMCs are therefore designated as myeloid-derived suppressor cells (MDSCs), and have been shown to accumulate in tumour-bearing mice and cancer patients. MDSCs are considered to be a strong contributor to the immunosuppressive tumour microenvironment and thus an obstacle for many cancer immunotherapies. Consequently, numerous studies are focused on the characterisation of MDSC origin and their relationship to other myeloid cell populations, their immunosuppressive capacity, and possible ways to inhibit MDSC function with different approaches being evaluated in clinical trials. This review analyses the current state of knowledge on the origin and function of MDSCs in cancer, with a special emphasis on the immunosuppressive pathways pursued by MDSCs to inhibit T cell functions, resulting in tumour progression. In addition, we describe therapeutic strategies and clinical benefits of MDSC targeting in cancer.

## Introduction

Myeloid cells play an important role in the innate immune response via the phagocytosis of pathogens (by macrophages), processing and presentation of antigens (by dendritic cells (DCs)), induction of an inflammatory response (by neutrophils), and promotion of wound healing (by platelets). Normally, the process of myelopoiesis involves the differentiation of multipotent progenitor cells and oligopotent myeloid precursors into unipotent monocytes, granulocytes, and DCs.^1^ Newly formed monocytes could further migrate into tissues where they differentiate into macrophages and DCs.^2^ Immature myeloid cells (IMCs), which represent myeloid progenitor cells and do not show immunosuppressive functions, are believed to be constantly present in healthy individuals. Chronic inflammatory conditions typical for cancers, chronic infections and autoimmune diseases were reported to impair IMC differentiation, supporting the accumulation of MDSCs.^3–5^

**Fig. 1:**
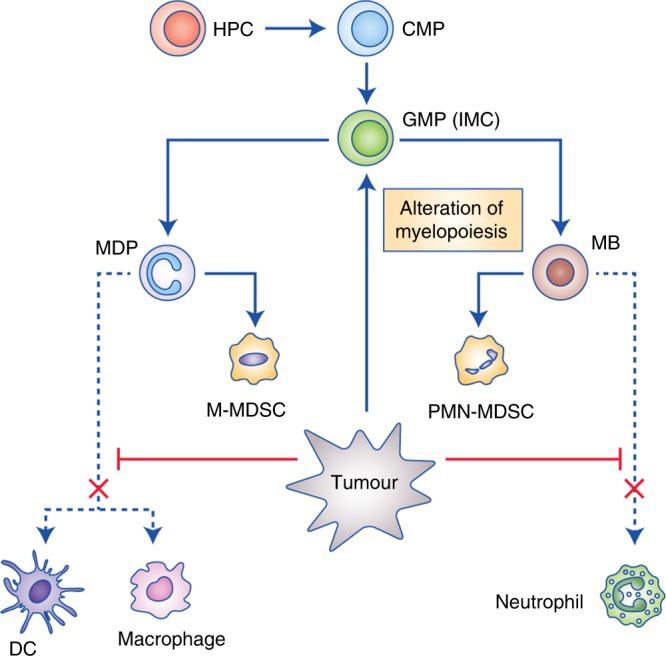
Myelopoiesis is altered under chronic inflammation. Under physiological conditions, hematopoietic progenitor cells (HPC) differentiate via common myeloid progenitor cells (CMP) into granulocyte/macrophage progenitor cells (GMP). These immature myeloid cells (IMC) further differentiate into monocytic/dendritic progenitor cells (MDP) or myeloblasts (MB) from which these cells further develop into dendritic cells (DCs)/macrophages or neutrophils, respectively. Under cancerous conditions, the tumour alters myelopoiesis in general and impairs further differentiation of progenitor cells, leading to the accumulation of monocytic myeloid-derived suppressor cells (M-MDSCs) and polymorphonuclear MDSCs (PMN-MDSCs)

Myelopoiesis can be disturbed by various conditions, such as inflammation. If inflammation is quickly resolved, then normal myelopoiesis can be restored; however, in the presence of a chronic inflammatory environment, the differentiation process of myeloid cells is impaired.^6^ In cases of chronic infection or cancer, a decrease in the amount of peripheral myeloid cells induces stronger myelopoiesis and increases the migration of cells before they have completed their differentiation process, which results in an accumulation of myeloid cells with strong immunosuppressive patterns and functions.^7–9^ Due to their function and myeloid origin, this heterogeneous cell population has been termed myeloid-derived suppressor cells (MDSCs) (Fig. 1 and Box 1).^10^ These cells represent a distinct population of IMCs that are being activated and expanded under chronic inflammatory conditions. Due to the high expression of immune checkpoint molecules, depletion of metabolites, promotion of other immunosuppressive cell populations, production of reactive radicals and immunosuppressive adenosine, these cells inherit powerful mechanisms to suppress the host’s immune system. Although MDSCs can also contribute to various aspects of tumour development, including angiogenesis and the formation of pre-metastatic-niches, this review summarises the current state of knowledge on the mechanisms of MDSC-mediated suppression of T cell functions, thereby promoting cancer progression.^11,12^

Box 1 Murine MDSCs are characterised by the co-expression of CD11b, an α-M integrin considered a pan-myeloid marker, and the myeloid differentiation antigen Gr1. The latter is a glycosylphosphatidylinositol-linked protein that consists of Ly6C and Ly6G subunits, allowing the differentiation between Ly6C^high^Ly6G^–^ monocytic (M-MDSCs) and Ly6C^low^Ly6G^+^ polymorphonuclear MDSCs (PMN-MDSCs).^124^ These subpopulations inherit features of monocytes and granulocytes, respectively, and are both capable of eliciting strong immunosuppressive functions. In humans, M-MDSCs are characterised as CD11b^+^CD14^+^HLA-DR^low/^^−^CD15^−^; and PMN-MDSCs as CD14^−^CD11b^+^ CD15^+^ (or CD66b^+^) cells.^125^ Lectin-type oxidised LDL receptor-1 (LOX-1) has also been proposed as a new marker to distinguish human PMN-MDSCs from non-immunosuppressive neutrophils.^104^ In addition, a subset of more immature human MDSCs defined as early-stage MDSCs (eMDSCs) lacks the expression of mature blood cell markers (including CD3, CD14, CD15, CD19, CD56) and are therefore characterised as Lin^–^HLA-DR^–^CD33^+^.^125^ By contrast, an eMDSC equivalent in mice has not been described.

## Expansion and recruitment of mdscs

Myelopoiesis has been shown to be altered at the stem-cell level in tumour-bearing mice, resulting in the accumulation of MDSCs accompanied by a low number of mature B cells.^13^ The role of B cells in tumour progression has been controversially discussed, describing both pro-and anti-tumour effects of these cells.^14^ The expansion of MDSCs is likely to be mediated by the same factors that regulate normal myelopoiesis, such as granulocyte-macrophage colony-stimulating factor (GM-CSF), granulocyte colony-stimulating factor (G-CSF), and macrophage colony-stimulating factor (M-CSF).^15,16^ Accordingly, *ex vivo* differentiation of murine IMCs into immunosuppressive MDSCs can be achieved through stimulation with GM-CSF and interleukin (IL)-6.^17^ IL-6 has been shown to promote the accumulation and immunosuppressive capacity of MDSCs mainly due to activation of the signal transducer and activator of transcription (STAT)3-signalling pathway, although the underlying molecular mechanisms are not completely understood.^18^ High levels of secreted of GM-CSF are common among different tumour entities and have been shown to induce the differentiation of MDSCs in mice with different transplantable tumours and with spontaneous breast tumours.^19,20^ In addition, GM-CSF blockade was able to abolish the immunosuppressive features of human MDSCs in vitro, highlighting GM-CSF as one of the main regulators of MDSC expansion.^21^

Various tumour-derived factors have also been shown to induce MDSCs in vitro, including prostaglandin E2 (PGE2), IL-6, IL-10, IL-1β, transforming growth factor (TGF)-β, as well as stem cell factor (SCF) and proangiogenic factors such as vascular endothelial growth factor (VEGF).^17^ Tumour cells are able to release these factors not only as soluble molecules but also entrapped within or bound to the surface of extracellular vesicles.^22^ Uptake of these vesicles containing PGE2 and TGF-β by bone marrow IMCs in vivo led to their conversion into immunosuppressive MDSCs.^22^ The induction of immunosuppression through tumour-derived extracellular vesicles seems to be an important mechanism of MDSC generation, as the pre-treatment of mice with these extracellular vesicles accelerates the formation of lung metastasis upon i.v. injection of tumour cells.^23^ The Toll-like receptor (TLR) signalling pathway appears to play a major role in this experimental setting, as this effect is not observed in the absence of MyD88, an important adaptor protein in TLR signalling.^23^ In addition, tumour extracellular vesicle-induced MDSCs from MyD88-deficient mice are less immunosuppressive than those from wild-type controls.^23^

**Fig. 2:**
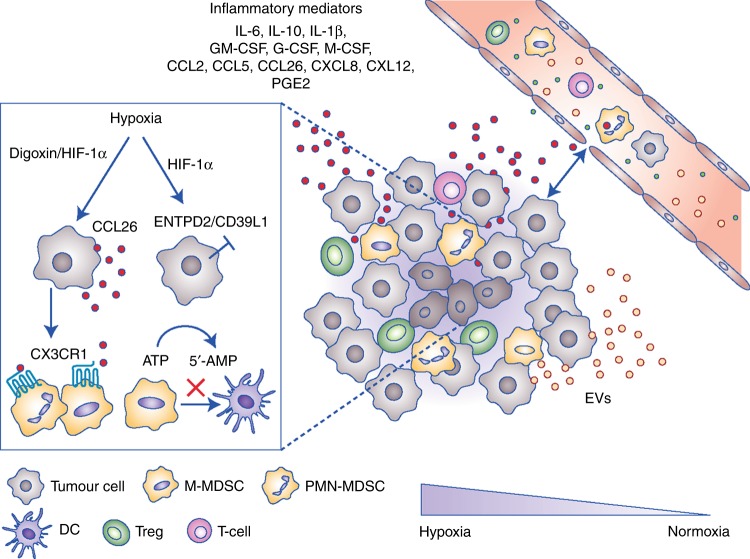
Myeloid-derived suppressor cells (MDSCs) are generated under chronic inflammatory conditions typical for cancer. Inflammatory factors that induce MDSC recruitment and expansion in the tumour microenvironment include interleukin (IL)-6, IL-10, IL-1β, granulocyte-macrophage colony-stimulating factor (GM-CSF), granulocyte colony-stimulating factor (G-CSF), macrophage colony-stimulating factor (M-CSF), chemokine (C-C motif) ligand 2 (CCL)2, CCL5, CCL26, chemokine (C-X-C motif) ligand 8 (CXCL)8, CXL12, and prostaglandin E2 (PGE2), released as soluble mediators or via extracellular vesicles (EVs). Hypoxia in the tumour microenvironment facilitates the expression of hypoxia-inducible factors digoxin and Hypoxia-inducible factor 1-alpha (HIF-1α) that induce the expression of the chemokine CCL26 and adenosine-producing ectoenzymes by tumour cells, leading to MDSC recruitment and accumulation

Various factors that accumulate in the tumour microenvironment (TME) in malignant diseases have been shown to contribute to the recruitment of MDSCs (Fig. 2). The expression of indoleamine 2,3-dioxygenase (IDO) by tumour cells, leading to the depletion of the essential amino acid tryptophan, was able to induce MDSC recruitment in mice, a process that was dependent on regulatory T cells (Treg).^24^ Since altered IDO expression has been associated with rapid tumour progression, IDO-mediated recruitment of MDSCs can play an important role in facilitating an immunosuppressive micromilieu.^25^

Hypoxia, which is commonly found in the TME, has also been recognised as in important factor in MDSC stimulation.^26–28^ Hypoxia-induced stabilisation of HIF-1 stimulated the expression of ectonucleoside triphosphate diphosphohydrolase 2 (ENTPD2/CD39L1), an ectoenzyme on MDSCs, leading to their accumulation.^27^ In a murine model of hepatocellular carcinoma (HCC), MDSC accumulation was described to be mediated by hypoxia-inducible factors (HIFs) such as digoxin and HIF-1, leading to the expression of the chemokine CCL26 on tumour cells and accumulation of MDSCs positive for the expression of CX3CR1, a CCL26 receptor in hypoxic tumour regions.^26^

Migration of MDSCs to the tumour site can also be mediated by various chemokines (Fig. 2).^29^ Studies have demonstrated an increase in the intratumoural expression of CCL2 in colorectal cancer patients, and a decrease in the numbers of MDSCs and immunosuppressive features of polymorphonuclear (PMN)-MDSCs, after CCL2 deletion in a spontaneous mouse model of colorectal cancer.^30^ Furthermore, CCL2 accumulation was found to correlate with poor prognosis in glioblastoma patients, whereas deficiency of CCL2 reduced the recruitment of monocytic (M)-MDSCs and Treg cells in a glioblastoma mouse model.^31^ CXC-motif chemokines have also been shown to contribute to MDSC recruitment, for example CXCL12 and IL-8 (CXCL8) have been reported to induce MDSC migration in the TME.^32,33^

In addition to the active recruitment of existing MDSCs, some cells can be converted into MDSCs. Adoptively transferred natural killer (NK) cells were shown to be converted in tumour-bearing mice by GM-CSF into CD11b^+^Gr1^+^ MDSCs, which expressed arginase-1 (ARG-1), produced reactive oxygen species (ROS) and exerted immunosuppressive activity.^34^

## MDSC immunosuppressive mechanisms

**Fig. 3:**
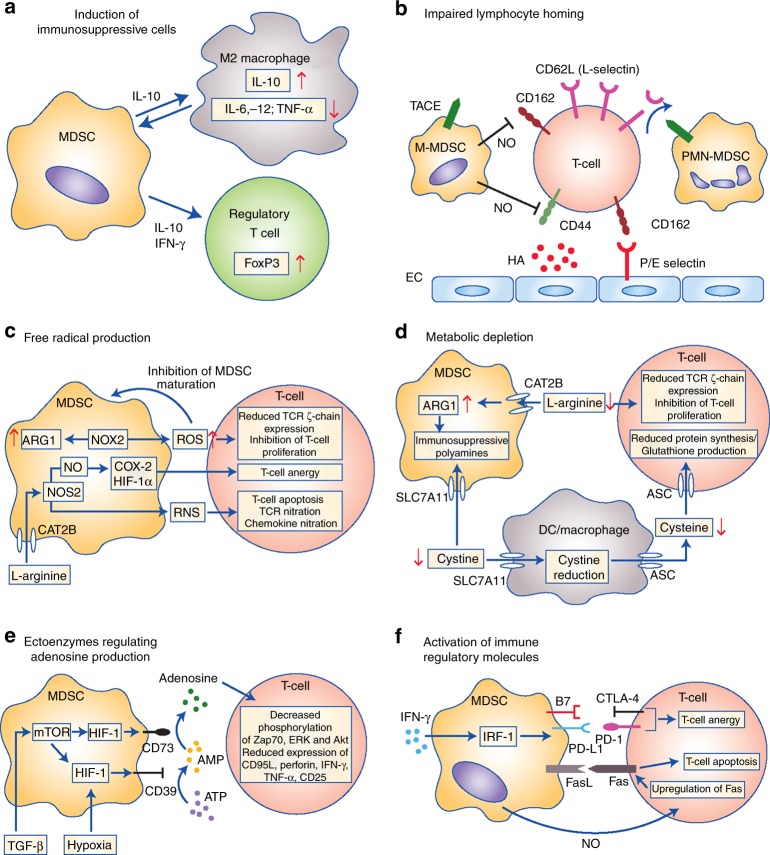
Main mechanisms of immunosuppression mediated by myeloid-derived suppressor cells (MDSCs). Mechanisms include the generation of immunosuppressive M2 macrophages and regulatory T cells via interleukin (IL)-10 and interferon (IFN)-γ secretion (**a**); impairment of lymphocyte adhesion to endothelial cells (ECs) and extravasation through nitric oxide (NO)-mediated downregulation of adhesion molecules CD162 and CD44, and tumor necrosis factor-alpha-converting enzyme (TACE)-mediated cleavage of CD62L (L-Selectin) on T cells (**b**); the production of reactive oxygen (ROS) and nitrogen species (RNS) through NADPH oxidase 2 (NOX-2) and nitric oxide synthase 2 (NOS2), leading to increased cyclooxygenase 2 (Cox-2), Hypoxia-inducible factor 1-alpha (HIF-1α) and arginase 1 (ARG1) expression and reduced T cell receptor (TCR) expression (**c**); the depletion and intracellular degradation of the amino acids L-arginine and cystine through increased uptake via the CAT2B and SLC7A11 transporters, respectively (**d**); induction of the ectoenzymes CD39 and CD73 via HIF-1 through transforming growth factor beta (TGF-β and hypoxic conditions, leading to adenosine production and reduced phosphorylation of extracellular signal–regulated kinase (ERK), protein kinase B (Akt) and Zap70, and reduced expression of CD95L, perforin, IFN-γ and tumour necrosis factor alpha TNF-α in T cells (**e**); and the expression of immune regulatory molecules B7, programmed death-ligand 1 (PD-L1) and FasL, causing T cell anergy and apoptosis via binding to their respective receptors (**f**)

MDSCs can display potent immunosuppressive and tumour-promoting functions in the TME via multiple mechanisms: induction of immunosuppressive cells, blocking of lymphocyte homing, production of reactive oxygen and nitrogen species, depletion of metabolites critical for T cell functions, expression of ectoenzymes that regulate adenosine metabolism, and expression of negative immune checkpoint molecules (Fig. 3).

### Induction of other immunosuppressive cells

MDSCs were shown to be able to induce the *de novo* generation of FoxP3^+^ Treg cells in vivo through a mechanism that was mediated by interferon (IFN)-γ and IL-10, but was independent of nitric oxide (NO) production.^35^ CD14^+^HLA-DR^–/low^ M-MDSCs isolated from patients with HCC could induce CD4^+^CD25^+^Foxp3^+^ Treg cells upon co-culture with autologous T cells.^36^ Furthermore, using a transplantable melanoma mouse model, it was demonstrated that Treg cells contribute to MDSC activity by inducing the expression of members of the B7 family of immune-regulatory ligands, including B7-H1 (also known as programmed cell death ligand 1 (PD-L1)), B7-H3 and B7-H4, as well as the production of IL-10 in these cells.^37^ Recruitment of CCR5^+^ Treg cells was also observed in two melanoma mouse models, which was induced by the CCR5 ligands CCL3, CCL4 and CCL5, produced by intratumoural M-MDSCs.^38^

M-MDSCs have also been described to secrete TGF-β and IL-10, which exert direct immunosuppressive effects on T effector cells or induce the generation of Treg cells.^35^ In addition, *ex vivo* differentiated murine MDSCs from wild-type mice showed increased expression of IL-10, TGF-β and iNOS when differentiated in the presence of TGF-β. In this study, TGF-β also induced the expansion of M-MDSCs, which have been shown to have a higher immunosuppressive capacity compared to PMN-MDSC. Therefore, TGF-β is considered to be involved in the generation of MDSCs and in the acquisition of their immunosuppressive pattern.^39^ In addition to Treg cell stimulation, MDSCs could shift macrophages to an M2-like phenotype with immunosuppressive features and low IL-12 production, thereby promoting tumour growth (Fig. 3a).^40^

### Blocking lymphocyte homing

Spleen MDSCs were reported to induce downregulation of the cell adhesion molecule L-selectin on CD4^+^ and CD8^+^ T cells, as well as on B cells in the spleen, leading to a reduction in the homing and antigen-dependent activation of CD8^+^ cells in lymph nodes.^41,42^ The downregulation of L-selectin on naïve T cells was found to be inversely correlated with MDSC levels in mice and is likely to be mediated by the expression of the metalloprotease ADAM 17 (TACE) on the surface of MDSCs (Fig. 3b).^43^ In addition, M-MDSCs have been found to counteract the activation-induced changes in CD44, L-selectin (CD62L) and CD162 expression by T cells in vitro.^44^ Downregulation of CD44, a receptor for the extracellular matrix component hyaluronic acid (HA), and CD162, a selectin P ligand, are believed to impair the extravasation and tissue infiltration of T cells. Induced downregulation of these molecules via M-MDSCs was found to be dependent on NO production (Fig. 3b).^44^

### Production of reactive oxygen and nitrogen species

MDSCs are well known to secrete reactive oxygen species (ROS), which are toxic to most cell types and thus contribute to the eradication of tumour-infiltrating lymphocytes. ROS include superoxide anions, hydroxyl radicals, hydrogen peroxide and singlet oxygen. The main pathway of ROS production in MDSCs involves the NADPH oxidase isoforms NOX1, NOX2, NOX3 and NOX4, which transfer electrons from NADPH to oxygen, creating superoxide radicals.^45^

Inhibiting the generation of ROS via the addition of catalase, an enzyme that detoxifies hydrogen peroxide, was shown to impair the immunosuppressive effect of MDSCs in vitro.^45^ MDSCs isolated from mice lacking NOX2 produced lower amounts of ROS and failed to inhibit the IFN-γ secretion and proliferation of antigen-specific CD8^+^ T cells.^46^ As well as their direct toxic effect towards tumour-directed immune cells, ROS also play a role in the expansion of MDSCs. Decreased ROS production resulted in the differentiation of MDSCs into F4/80^+^ Gr1^–^ macrophages or CD11c^+^CD11b^+^ DCs, indicating a role for NOX2 in the maintenance of the MDSC population (Fig. 3c).^46^

Augmented ROS levels also stimulated the expression of VEGF receptors on MDSCs, contributing to MDSC recruitment into the TME.^47^ In a spontaneous melanoma mouse model, inducible NO synthase (iNOS)-dependent production of VEGF was identified as a key regulator of intratumoural MDSC accumulation.^48^ IMCs from wild-type mice showed a high expression of both VEGFR1 and VEGFR2 after treatment with tumour cell-conditioned medium, indicating a prominent role of these receptors in the accumulation and recruitment of MDSCs.^48^ This assumption was supported by the finding of increased expression of VEGFR1 and 2 on intratumoural MDSCs from a mouse ovarian cancer model, which was associated with their accumulation at the tumour site.^49^ VEGF-mediated activation of the transcription factor STAT3 is assumed to be the main mechanism of VEGF-mediated MDSC activation.^50,51^ Interestingly, STAT3 activation induced VEGF expression, which could create a positive feedback loop.^52,53^

MDSCs themselves were found to be partially protected from the detrimental effects of ROS through the expression of the Nrf2 transcription factor, an important mediator of the cellular antioxidant response.^54^ Tumour-derived MDSCs from Nrf2^–/–^ mice displayed an increased level of apoptosis, reduced secretion of hydrogen peroxide and increased levels of oxidative stress, indicating a role for Nrf2 in promoting MDSC survival and function.^54^ In addition, MDSCs are characterised by a distinct metabolic programme with increased glycolysis, which leads to the intracellular accumulation of the anti-oxidative intermediate phosphoenolpyruvate (PEP).^55^ As a result, increased rates of glycolysis were shown to prevent ROS-induced apoptosis of MDSCs and promote their accumulation in vivo.^55^ Furthermore, scavenging of ROS leads to the *ex vivo* increased differentiation of IMCs isolated from tumour bearing mice into DCs and macrophages, indicating an influence of ROS in maintaining the MDSC population.^56^

In addition to producing ROS, MDSCs produce high levels of reactive nitrogen species (RNS), predominantly nitric oxide (NO), via the activation of iNOS.^57^ Accumulating NO levels were demonstrated to strongly induce the expression of cyclooxygenase 2 (COX-2) and HIF-1α.^41,42^ Together with COX-1 and prostaglandin synthases,^58,59^ COX-2 regulates the production of PGE2.^60^

PGE2 has been shown to induce the upregulation of IDO, IL-10, ARG-1 and other immunosuppressive markers in *ex vivo*-generated MDSCs.^61^ In addition, recent studies linked PGE2 to the overexpression of DNA methyltransferase 3 A (DNMT3A) in MDSCs, resulting in the activation of these cells.^62^ Enhanced HIF-1α expression stimulated the production of VEGF, which is not only important for angiogenesis but has also been shown to inhibit the differentiation of DCs and to induce Treg cell accumulation.^63^

In the presence of low L-arginine levels, which can occur due to metabolite depletion by MDSCs, iNOS was demonstrated to stimulate the production of peroxynitrites (ONOO_2_), highly reactive radicals that can cause T cell apoptosis, as well as nitration of the T cell receptors (TCRs), thereby blocking T cell activation.^64^ RNS in the TME were shown to induce the nitration of chemokines such as CCL2, which could inhibit the recruitment of tumour-reactive lymphocytes.^65^ Interestingly, the migration of MDSCs, which is partially dependent on CCL2, was not affected by CCL2 nitration.^65^ Further studies revealed an inhibitory effect of iNOS-dependent NO production in MDSCs on different FcR-mediated functions of NK cells.^66^ Adoptive transfer of MDSCs in a murine pancreatic cancer model impaired NK cell-mediated antibody-dependent cytotoxicity, cytokine production, and signal transduction, leading to impaired efficacy of monoclonal antibody therapy.^66^

Another molecule that is believed to contribute to free radical production in MDSCs is myeloperoxidase (MPO), an enzyme that is highly abundant in neutrophils and PMN-MDSCs, inducing cytotoxicity during the respiratory burst.^67^ Splenic PMN-MDSCs from tumour-bearing mice displayed an increased activity of MPO, ARG-1 and ROS producing enzymes, which correlated with their ability to suppress antigen-specific T cell responses in vitro.^67^ In this setting, PMN-MDSCs expressed high levels of CD115 and CD244 compared to splenic neutrophils from wild-type controls. In addition, the MPO level have been shown to be increased in the plasma of renal cell carcinoma patients, presumably produced by PMN-MDSC.^68^

### Depletion of metabolites critical for T cell functions

MDSCs can decrease the availability of metabolites and factors, such as L-arginine, that are crucial for the function of the mammalian immune system. L-arginine is the substrate for four different enzymes expressed in MDSCs as different isoforms of nitric oxide synthases (NOS1, NOS2, and NOS3), arginases (ARG-1 and ARG–2), arginine-glycine amidinotransferase, and L-arginine decarboxylase. Whereas NOS catalyse the conversion of L-arginine to NO and l-citrulline, arginases support the reaction of L-arginine to L-ornithine and urea.^69^ L-ornithine can be further metabolised to L-proline, an important precursor for collagen synthesis and immunosuppressive polyamines. ARG-1 upregulation in MDSCs leads not only to the inhibition of T cell functions, but also contributes to the production of extracellular matrix components and therefore tissue remodelling and tumour growth.^69^ The expression of ARG-1 by MDSCs can be induced by the Th2 cytokines IL-4, IL-10 and IL-13. By contrast, expression of iNOS is mainly regulated by the Th1 cytokines IFN-γ, TNF-β and TNF-α.^69^ In addition, activation of TLRs through lipopolysaccharide has also been shown to induce both ARG-1 and iNOS expression.^69^

MDSCs have also been reported to deplete L-arginine from the TME through increased uptake mediated by the CAT-2B transporter, followed by L-arginine degradation mediated by increased ARG-1 expression in these cells.^70^ The lack of extracellular L-arginine could inhibit the proliferation of activated T cells and reduce the expression of the TCR-ζ chain (Fig. 3d).^70^ In this case, the reduced expression of the TCR-ζ chain is probably due to a shorter half-life span of the TCR-ζ chain mRNA, since this mechanism has been shown to be present in Jurkat T cells in vitro.^71^ Interestingly, a recent paper reported that ARG-1 expression is not crucial for MDSC-mediated immunosuppression, although ARG-1 expression could be induced by activated T cells.^72^ In this setting, direct cell-cell contact was necessary for MDSCs to inhibit T cell proliferation. Thus, the authors concluded that soluble factors play only a secondary role in the inhibition of T cell proliferation, compared with surface molecules such as PD-L1.^72^ Since numerous studies demonstrated a contribution of ARG-1 to MDSC function, further investigations will be necessary to define its exact role in MDSC function.

Due to its ability to form disulphide bonds, cysteine is known as an important prerequisite for protein biosynthesis. Most mammalian cells can synthesise cysteine from intracellular methionine using the enzyme cystathionine γ-lyase, or can import oxidised cysteine (cystine) via the SLC7A11 cysteine/glutamate antiporter.^73^ However, as T cells lack cystathionine and do not express cystine transporters, cysteine is considered as an essential amino acid for T cells.^74^ Macrophages and DCs supply T cells with cysteine by taking up cystine through their SLC7A11 transporters, reducing cystine to cysteine intracellularly, and finally secreting cysteine into the extracellular space through alanine–serine–cysteine (ASC) transporters. Thereby, T cells are enabled to take up cysteine via their ASC transporters. MDSCs have been shown to express the SLC7A11 transporter but not the ASC transporter, enabling them to deplete cystine without secreting cysteine, thereby impairing T cell functions (Fig. 3d).^75^ As cysteine is also involved in the generation of glutathione, an antioxidative molecule protecting cells from free radicals including ROS, MDSCs can also impair the resistance of immune cells to ROS.^76^

### Expression of ectoenzymes regulating adenosine metabolism

Another mechanism used by MDSCs to inhibit T cell functions includes the generation of adenosine from ATP.^77^ Ectonucleoside triphosphate diphosphohydrolase 1 (E-NTPDase1, CD39) is known to convert ATP released into the extracellular space into AMP, before ecto-5’-nucleotidase (Ecto5’NTase, CD73) catalyses its dephosphorylation into adenosine. Extracellular adenosine was demonstrated to inhibit priming of naïve T cells by preventing phosphorylation of Zap70, ERK and Akt,^78^ as well as reducing the expression of effector molecules on activated T cells such as CD95L, perforin, IFN-γ, TNF-α and CD25 (Fig. 3e).^79^ Furthermore, it was recently reported that tumour-derived TGF-β induced the expression of CD39 and CD73 on MDSCs isolated from the peripheral blood and tumours of patients with non-small cell lung cancer in a HIF-1α-dependent manner, resulting in the accumulation of immunosuppressive adenosine.^77^ In line with these findings, activation of AMP-activated protein kinase α (AMPKα) through administration of the drug metformin downregulated the expression of HIF-1, CD39 and CD73 in MDSCs, which was associated with a longer overall survival in patients with ovarian carcinoma, alongside a decrease in the number of circulating CD39^+^CD73^+^ MDSCs and enhanced anti-tumour activities of circulating CD8^+^ T cells.^80^ These findings implicate ectoenzymes as potential new therapeutic targets in cancer treatment.

### Expression of negative immune checkpoint molecules

PD-L1 is known to be a prominent negative regulator of T cell functions and a mediator of immune evasion by tumour cells.^81^ Inhibition of signalling by PD-L1 or another immune-checkpoint component, CTLA-4, has proven to be beneficial for cancer patient survival. The success of this approach seems to be dependent on T cells infiltrating the tumour, and therefore works better in so-called ‘hot’ tumours, such as malignant melanoma, which show a strong infiltration with immune cells.^82,83^ PD-L1 exerts its effect by binding to its receptor PD-1 on T cells, inducing T cell anergy and apoptosis. Not surprisingly, PD-L1 expression on MDSCs has been shown in various reports to be a potent mediator of immunosuppression and to be increased in cancer patients and tumour-bearing mice, compared with healthy controls.^84,85^ Not only has the number of MDSCs been shown to be increased in tumour tissue from patients with non-small cell lung cancer, but tumour-associated PMN-MDSCs were found to express higher levels of PD-L1 than their circulating counterparts.^84^ PD-L1 expression on PMN-MDSCs was also found to be higher among non-responding ipilimumab-treated melanoma patients, compared to responding patients.^86^ Furthermore, low MDSC levels prior to treatment with ipilimumab, which blocks CTLA-4, were associated with better survival in advanced melanoma patients, indicating a possible function for MDSCs as a predictive biomarker for therapy with immune checkpoint inhibitors.^87^ The induction of PD-L1 on IMC has been recently shown to be mediated by soluble factors M-CSF and VEGF ex vivo.^88^ In a murine model of colorectal cancer, PD-L1 induction in MDSCs could be significantly decreased after the neutralisation of IFN-γ.^85^

## Signalling pathways important for mdsc-mediated immunosuppression

The immunosuppressive phenotype of MDSCs relies on the activation of different intracellular signalling pathways, which are often stimulated through the interaction of MDSCs with immune cells.

In this context, the Janus kinase (JAK)–STAT signalling pathway is of special interest. Activation of chemokine, cytokine or growth factor receptors by their cognate ligands induces the recruitment and stimulation of JAK, followed by activation of STAT proteins.^89^ In the context of MDSC activation, STAT3 and STAT1 are considered to be the major contributors to immunosuppressive mechanisms. Activated T cells were reported to secrete IL-10, which induces PD-L1 expression on MDSCs in a STAT3-dependent manner.^90^ Furthermore, STAT3 activation could induce VEGF production in MDSCs, whereas STAT3 inhibition by the tyrosine-kinase inhibitor sunitinib reduced tumour angiogenesis and MDSC expansion in vivo.^91^ As mentioned above, VEGF-mediated STAT3 activation leads to further secretion of VEGF and expression of VEGF receptors, thereby supporting MDSC accumulation and tumour growth.^50–53,91^

STAT3 also directly regulates the expression of NOX2 and the calcium-binding pro-inflammatory proteins S100A9 and S100A8.^46,92^ These proteins were shown to activate signalling through nuclear factor κB (NF-κB) in MDSCs and to contribute to the production of ROS.^92,93^ Activation of the NF-κB pathway is known to be a potent inducer of COX-2 expression, which ultimately leads to the production of PGE2.^94^ Increased COX-2 mRNA expression correlates positively with ARG-1 and NOS2 transcript levels in tumour-infiltrating MDSCs, and thus a regulatory function for PGE2 on the expression of these immunosuppressive proteins may be assumed.^6^ Furthermore, PGE2 has been shown to induce the generation of MDSCs from murine bone marrow stem cells, which is at least partially mediated through the activation of the PGE2 receptor EP2.^95^

The expansion and activation of MDSCs was found to be mediated by STAT1–IFN-γ-dependent signalling, which has been shown to be involved in the upregulation of the anti-apoptotic protein Bcl2a1.^96^ Activation of STAT6 through the binding of IL-4 or IL-13 to IL-4Rα induced an immunosuppressive pattern of MDSCs, as reflected by the expression of ARG-1 and TGF-β.^97^ Furthermore, the upregulation of PD-L1 expression on MDSCs through IFN-γ was demonstrated to be mediated by subsequent activation of STAT1 and interferon regulatory factor 1 (IRF1).^85^ Finally, the pro-inflammatory cytokine IL-6, which is produced by various tumours, was found to be involved in the expansion and activation of MDSCs via inhibition of the suppressor of cytokine signalling 3 (SOCS3) protein, leading to phosphorylation of JAK1, JAK2, TYK2, STAT1 and STAT3 proteins.^18^

## Relation of mdscs to immunosuppressive neutrophils

Recent studies have indicated that neutrophils can also promote tumour progression and metastasis formation under specific conditions through the induction of angiogenesis and an immunosuppressive environment.^98^ PMN cells have gained increasing interest over the past decade due to accumulating evidence indicating that tumour-associated neutrophils promote tumour growth, and the fact that PMN-MDSCs are the prominent subtype of MDSC in most murine tumour models and human cancers.^99,100^

The discrimination between PMN-MDSCs and neutrophil subpopulations is still a subject of debate. Some studies suggest that PMN-MDSCs represent a group of pathologically activated neutrophils, also termed N2 neutrophils.^101^ These cells can elicit powerful tumour-promoting mechanisms, including upregulation of ARG-1 expression and angiogenesis as well as the stimulation of metastasis formation. By contrast, N1-type neutrophils display functions of classical neutrophils like phagocytosis, antibody-dependent cytotoxicity and recruitment of leukocytes.^102^ It is hypothesised that N2 neutrophils are either recruited peripheral PMN-MDSCs or peripheral-blood-derived neutrophils that acquire an N2 phenotype under the influence of TGF-β in the TME.^103^ LOX-1 has been identified as a marker of human immunosuppressive PMN-MDSCs that can distinguish them from non-suppressive neutrophils, although the LOX-1 equivalent in mice was not found to be sufficient to distinguish these cells from classical neutrophils.^104^

In peripheral blood, neutrophils can be separated, based on their density, into tumour-promoting low-density neutrophils (LDNs) and high-density neutrophils (HDNs), which represent classical innate immune cells.^105^ Although LDNs could not be detected in the peripheral blood of healthy subjects, this population was found to be expanded in cancer patients and tumour-bearing mice.^105^ It has been proposed that the LDN fraction consists of mature N2-neutrophils and immature PMN-MDSCs.^105^

## MDSC targeting in cancer therapy

Increasing numbers of preclinical and clinical studies have been performed over past years, that have sought to evaluate the safety and efficacy of MDSC inhibition alone or in combination with radiotherapy, chemotherapy, surgery or different kinds of immunotherapy to target cancers. Current treatment strategies aim to deplete MDSCs, inhibit their immunosuppressive potential, block their recruitment to the tumour site, or to modulate myelopoiesis.^106^

Treatment of pancreatic cancer patients with the chemotherapeutic drug gemcitabine reduced numbers of PMN-MDSCs and Tregs.^107^ In addition, immunotherapeutic treatment approaches with IL-2 and anti-CD40 antibody sensitised MDSCs to Fas-mediated apoptosis in different murine tumour models, highlighting the possible use of existing therapies to efficiently deplete MDSC in cancer patients.^108^ Treatment with an agonist of TLR8, which is expressed on M-MDSCs but not PMN-MDSCs, has been reported to induce Fas–FasL-dependent apoptosis and to restore IL-2 secretion by T cells activated via CD3–CD28.^109^

Blocking the immunosuppressive function of MDSCs can be achieved by targeting phosphatidylinositol 3-kinase (PI3K)δ and PI3Kγ. PI3K activation leads to the inhibition of NF-κB and activation of C/EBPβ, thereby initiating an immunosuppressive transcriptional program.^110^ Knockout of PI3K was found to reduce the accumulation of PMN-MDSC in tumour-bearing mice, breaking immune tolerance to cancer.^111^ Inhibition of both isoforms of this kinase delayed tumour growth and prolonged survival in tumour models of head and neck cancer, when used in combination with a PD-L1 blocking antibody, indicating a beneficial effect of MDSC inhibition in combination with immune-checkpoint inhibition.^112^

Targeting PD-L1 or CTLA-4 has led to durable responses in different cancer entities, but only in a subset of patients. Metastatic castration-resistant prostate cancer, for example, shows a strong de novo resistance to CTLA4 blockade.^113^ In a newly developed chimeric mouse model of prostate cancer, combined treatment with PD-L1 inhibition as well as multi-kinase inhibitors cabozantinib and BEZ235, which induce a decrease in MDSC function, proved to be considerably more effective than treatment with a single agent.^114^ This highlights the necessity of including MDSC neutralisation in novel strategies of combined cancer treatment. The recently described modulating effects of the diarylheptanoid curcumin on STAT3 and JAK2 signalling, leading to a decreased production of IL-6 in MDSCs, could also be beneficial in combined therapies while eliciting no adverse effects.^115^ Several attempts to apply STAT3 inhibitors in clinical studies for targeting tumour-associated myeloid cells were hindered by the unexpected adverse effects and limited efficacy of these compounds.^116^ More recent approaches aim to interfere with STAT3 mRNA by the administration of siRNA or decoy oligonucleotides. For example, AZD9150 is a STAT3 oligonucleotide inhibitor that is currently under investigation in combination with immune-checkpoint inhibitors in phase I/II clinical trials.^106^

Mobilisation of MDSCs from the bone marrow has been shown to be inhibited through the administration of bisphosphonates, drugs that are used to prevent bone loss in cancer patients with bone metastases.^117^ Bisphosphonates can prevent prenylation of matrix metalloproteinases (MMPs) from undergoing prenylation, a post-translational modification that is essential for their function. As a result of reduced MMP9 prenylation, cleavage of the tyrosine kinase c-Kit is diminished, causing reduced mobilisation of MDSCs and reduced VEGF release.^117^ Most MMP inhibitors failed in different clinical trials, showing severe side effects that hamper their usage to inhibit MDSCs mobilisation in cancer.^118^ Amino-bisphosphonates show a good safety and tolerance and seem to exert therapeutic effects, making them promising candidates to target MDSCs.^119–121^

Other therapeutic approaches are aimed at promoting MDSC differentiation, which can be achieved by all-trans-retinoic acid (ATRA). ATRA was reported to induce the rapid differentiation of MDSCs into macrophages and DCs, which was associated with the reduction of ROS production, via the activation of the ERK1/2 kinase pathway.^122^ In addition, ATRA enhanced the immune response to vaccination with DCs in small-cell lung cancer patients by effectively depleting MDSCs.^123^ Furthermore, pharmacological inhibition of the ATP-converting ectoenzyme ENTPD2 was reported to promote MDSC differentiation, to delay the growth of HCC, and to contribute to the beneficial effects of immune checkpoint inhibition.^27^

## Conclusion

Numerous publications have documented a pivotal role for highly immunosuppressive MDSCs in tumour progression in mice and cancer patients. This heterogeneous population of IMCs, which is generated and activated under chronic inflammatory conditions and accumulates in the TME, represents one of the major hurdles for efficient cancer immunotherapy. Accordingly, inhibition of MDSCs in cancer therapy has proven to be a potentially promising and well-tolerated treatment. As the enrichment and activation of MDSCs seem to be a general characteristic of malignant diseases, targeting these cells could be applied to treat various tumour entities. It is therefore critically important to combine the neutralisation of different MDSC functions with current treatment strategies to increase the efficacy of these therapies. However, despite promising preclinical data, more clinical studies are needed to demonstrate the synergistic effects of inhibiting MDSC mobilisation and functions in conjunction with existing immunotherapies.
